# Gut microbial diversity moderates polygenic risk of schizophrenia

**DOI:** 10.3389/fpsyt.2024.1275719

**Published:** 2024-02-01

**Authors:** Liyuan Zhang, Xiuxia Yuan, Xue Li, Xiaoyun Zhang, Yiqiao Mao, Shaohua Hu, Ole A. Andreassen, Yunpeng Wang, Xueqin Song

**Affiliations:** ^1^Department of Psychiatry, The First Affiliated Hospital of Zhengzhou University, Zhengzhou, China; ^2^Henan International Joint Laboratory of Biological Psychiatry, Zhengzhou University, Zhengzhou, China; ^3^Henan Psychiatric Transformation Research Key Laboratory, Zhengzhou University, Zhengzhou, China; ^4^School of Information Engineering, Zhengzhou University, Zhengzhou, China; ^5^Department of Psychiatry, The First Affiliated Hospital, Zhejiang University School of Medicine, Hangzhou, China; ^6^Norwegian Centre for Mental Disorders Research (NORMENT), Institute of Clinical Medicine, Oslo University Hospital, University of Oslo, Oslo, Norway; ^7^Centre for Lifespan Changes in Brain and Cognition (LCBC), Department of Psychology, University of Oslo, Oslo, Norway

**Keywords:** schizophrenia, microbiota, polygenic score, α-diversity, *Romboutsia*

## Abstract

**Background:**

Schizophrenia (SCZ) is a heritable disorder with a polygenic architecture, and the gut microbiota seems to be involved in its development and outcome. In this study, we investigate the interplay between genetic risk and gut microbial markers.

**Methods:**

We included 159 first-episode, drug-naïve SCZ patients and 86 healthy controls. The microbial composition of feces was characterized using the 16S rRNA sequencing platform, and five microbial α-diversity indices were estimated [Shannon, Simpson, Chao1, the Abundance-based Eoverage Estimator (ACE), and a phylogenetic diversity-based estimate (PD)]. Polygenic risk scores (PRS) for SCZ were constructed using data from large-scale genome-wide association studies. Effects of microbial α-diversity, microbial abundance, and PRS on SCZ were evaluated via generalized linear models.

**Results:**

We confirmed that PRS was associated with SCZ (OR = 2.08, *p* = 1.22×10^−5^) and that scores on the Shannon (OR = 0.29, *p* = 1.15×10^−8^) and Simpson (OR = 0.29, *p* = 1.25×10^−8^) indices were inversely associated with SCZ risk. We found significant interactions (*p* < 0.05) between PRS and α-diversity indices (Shannon, Simpson, and PD), with the effects of PRS being larger in those exhibiting higher diversity compared to those with lower diversity. Moreover, the PRS effects were larger in individuals with a high abundance of the genera *Romboutsia*, *Streptococcus,* and *Anaerostipes* than in those with low abundance (*p* < 0.05). All three of these genera showed protective effects against SCZ.

**Conclusion:**

The current findings suggest an interplay between the gut microbiota and polygenic risk of SCZ that warrants replication in independent samples. Experimental studies are needed to determine the underpinning mechanisms.

## 1 Introduction

Schizophrenia (SCZ) is a severe mental disorder characterized by psychotic symptoms and is associated with impaired cognitive functions and social disability (1). The lifetime prevalence of SCZ is estimated to be around 1%, and approximately 24 million people are affected by SCZ worldwide (2, 3). Despite the heavy disease burden, the etiology of SCZ is largely unknown (1). Twin and family studies have estimated a heritability of 0.6–0.8 (4, 5), indicating that genetic variation may contribute substantially to the disease risk. Recent large genome-wide association studies (GWAS) have identified over 200 risk loci associated with SCZ (6, 7). Surprisingly, only 2.4% of the variation in degree of liability to SCZ can be explained by genome-wide significant variants; when genome-wide common variants are aggregated into polygenic risk scores (PRS), these scores could explain up to 20% of the risk for SCZ in independent samples. The gap between the PRS-explainable variation and the estimated heritability for SCZ could be explained by several factors, for example, ancestrally unmatched GWAS samples from the prediction sample (8) and rare variants missed by GWAS (9). Of note is that gene–environmental interactions may contaminate heritability estimates (9–11).

Research on the environmental influences on SCZ has a long history and has identified an array of risk factors, including living in urban regions, migration, childhood adverse events, birth complications, and cannabis and other substance use (12–14). Studies of genetics-by-environment effects on SCZ have been predominantly based on the candidate-gene design. For instance, these studies have suggested that cannabis use may interact with the *DRD2* (15) and *AKT1* (16) genes in conferring risk for SCZ, while the *FOXP2* gene might moderate the effects of childhood emotional abuse on SCZ (17). However, the reliability of these and other candidate-gene-based findings has been challenged by recent GWAS results (18–20). Recent work has advocated the use of PRS derived from large-scale GWAS for investigating gene–environment interplay in psychiatric disorders (21). In addition, environmental factors are complex to measure and interdependent, and previous work has focused only on one or few factors per study (22). Identifying novel modifiable and “aggregated” environmental exposure factors would be beneficial in gene–environment interaction research on SCZ.

The gut microbiome has become a burgeoning field in medical research (23); features of this have been frequently linked to the risk and treatment outcomes of SCZ (24–31). The gut microbiota composition is typically characterized by the relative abundance of its constituent microbes on multiple taxonomic levels, e.g., phylum, family, or genus. It can also be described by global measures, the α-diversity and β-diversity (32). The α-diversity characterizes the within-sample distributions of constituent microbes, for example, by the richness and evenness of the distribution; the β-diversity describes between-group differences in microbial distributions, for example, between SCZ patients and healthy controls. While several significant associations of taxon and global measures with SCZ have been reported, these findings are not consistent, as reported by recent reviews and meta-analyses (33–36). These studies have also shown that α-diversity is not statistically associated with SCZ, but with some evidence an association for first-episode psychosis patients; β-diversity has been found to be significantly associated with psychosis and SCZ (37, 38). On the taxon level, there are only two genera, *Anaerotruncus* and *Lactobacillus,* from the phylum Firmicutes, whose relative abundance has been found to be associated with SCZ in two or more independent studies. On the one hand, these early findings call for more studies to corroborate existing evidence; on the other hand, strategies to overcome the large degrees of heterogeneity in previous studies are imperative. The identification of factors that interact with microbiota composition and SCZ may be one such strategy.

Here, we present the first study investigating the interactions between gut microbial composition and host genome in relation to the risk of SCZ. To reduce confounding, we recruited 159 first-episode drug-naïve SCZ patients and 86 demographically matched healthy controls (matched on age, sex, years of education, smoking habits, and BMI) from China. We investigated the effects of PRS, which measures an individual’s liability to SCZ, on five widely studied measures of the α-diversity of the gut microbiota (32, 35, 39)[ACE (40) and Chao1 (41) as measures of richness; Simpson (42) and Shannon (42) as measures of evenness; and phylogenetic diversity (PD) (43)] and their interactions. Furthermore, we tested for interaction effects between PRS and specific taxa in terms of the effect on SCZ. Finally, we tested whether our results varied when using PRS constructed from European vs. East-Asian sample-based GWAS.

## 2 Materials and methods

### 2.1 Participants and clinical assessment

Two hundred and eleven first-episode drug-naïve SCZ patients and 145 demographically matched healthy controls (HCs) were enrolled at the Department of Psychiatry of the First Affiliated Hospital of Zhengzhou University, China. This study was approved by the Human Ethics Committee of the hospital (No. 2016-LW-17). All participants provided signed informed consent.

Patients were diagnosed by two trained psychiatrists at the department using the Diagnostic and Statistical Manual of Mental Disorders, fourth edition (DSM-IV) criteria obtained via the Structured Clinical Interview for DSM-IV (SCID) (44). Severity of psychosis was evaluated with the Positive and Negative Syndrome Scale (PANSS) (45), and a general clinical assessment was performed, including height and weight for calculation of body mass index (BMI). At the hospital visits, age, sex, history of smoking, and years of education were also collected for all participants.

On enrollment, participants were required to be free of previous treatments for any mental disorders, free of diagnoses with organic diseases (46), not pregnant or lactating, and free of any use of antibiotic or anti-inflammatory agents during the prior month; not to have have a BMI >28 kg/m^2^; and not to have a total PANSS score ≤ 60. Other than the PANSS score, the same exclusion criteria were applied to HCs.

### 2.2 Fecal sample collection and processing

Fresh fecal samples were collected from all participants between 8 a.m. and 9 a.m. and immediately stored in a freezer at −80°C for gut microbiota assay. Whole blood samples were also collected at the same time for genotyping. A fecal sample of 0.2 g was used to extract the microbiome DNA using the Cetyl Trimethyl Ammonium Bromide (CTAB)/SDS procedure (47). The extracted DNA sample was diluted to 1 ng/ul with sterile water before sequencing. The 16S rRNA gene of distinct regions (V3–V4) was amplified using a specific primer (i.e., 16S V4: 515F-806R) with barcodes. PCR was performed in reaction volume of 30 μL with 15 μL of Phusion^®^ High-Fidelity PCR Master Mix (New England Biolabs), 0.2 μM of forward and reverse primers, and 10 ng of template DNA. The obtained PCR products were purified using a GeneJETTM Gel Extraction Kit (Thermo Scientific^TM^, USA) following the manufacturer’s protocol. Purified PCR products were sequenced using the Ion S5 TM XL platform, which generates DNA 400–600 bp long single-end reads. These raw DNA sequences were quality-controlled using the Cutadapt program (V1.9.1) (48). The outputs from Cutadapt were aligned to the SILVA reference database with default settings, and chimera sequences (49) were removed using UCHIME (50).

The UPARSE program (V7.0.1001) (51) was used to assign quality-controlled sequences to operational taxonomic units (OTUs) with a sequence similarity threshold of 97%. Based on the assigned OTUs, the five most widely used α-diversity indices were estimated by QIIME (52): Shannon (42), Simpson (42), the Abundance-based Coverage Estimator (ACE) (40), Chao1 (41), and Faith’s phylogenetic diversity metric (PD) (43). Since these indices are highly skewed, all indices (except Shannon) were transformed and standardized to a mean of 0 and standard deviation of 1. For the ACE, Chao1, and PD indices, the natural log transformation was further applied. For the Simpson index, which falls within the range of 0–1, the logit transformation was performed. For the Shannon index, no transformation was applied.

### 2.3 Genotyping and processing

DNA was extracted from whole blood samples and was genotyped using the InfiniumOmniZhongHua_8v Array (Illumina, Inc.) on an iScan instrument at Bio Miao Biological Technology (Beijing). Genotype calling and quality control (QC) were performed using GenomeStudio V2011. Quality assessments resulted in the exclusion of 23 samples from subsequent analyses due to a low call rate, i.e., <95%. The resultant file was exported to PLINK (53) format from the GenomeStudio platform. The following PLINK parameters were used for further QC before imputation: *−maf 0.01*, *−mind 0.1*, *−geno 0.05*, and *–hwe 0.0001*. In addition, variant strands were checked with the command *–flip*, and the concordance between self-reported and genetically predicted sex was checked with the command *–check-sex*. These pre-imputation high-quality data were also used for checking relatedness between subjects after construction of a thinned dataset via PLINK with the command *–indep-pairwise 1,500 150 0.2 –maf 0.05*, followed by *–Z-genome –min 0.06*. After QC, 287 subjects remained. Among these subjects, gut microbiota data were available for 159 patients and 86 HCs. The program genipe (54) was used to call IMPUTE2 (55) and Shapeit2 (55) for imputation of our sample to the 1,000 Genomes samples (56). For post-imputation QC, single nucleotide polymorphisms (SNPs) with imputation r^2^ < 0.8, MAF <0.05, or Hardy–Weinberg test <1×10^−6^ were removed. In total, 4,896,670 SNPs remained. These remaining SNP dosages were hard-coded to the best-guess genotype, i.e., 0, 1, or 2 copies of the reference allele.

Subtle population structures within our sample were estimated using the principal component analysis (PCA) methods implemented in PLINK. Before PCA, pre-imputation quality-controlled genotypes were further processed using the PLINK commands *–maf 0.1* and *–indep-pairwise 100 50 0.1*. The remaining SNPs were included in the PCA estimation with the PLINK command *–pca*. Since the eigenvalue scree plot showed a very flat pattern, we included the top 10 principal components (PC1-10) in our analysis.

### 2.4 Polygenic risk scores

The imputed best-guess genotypes were used for construction of polygenic risk scores. SCZ GWAS summary statistics for both European and East Asian populations were downloaded from https://www.med.unc.edu/pgc/download-results/scz/ with permission. After filtering out SNPs that did not exist in our data from the summary statistics, the program PRC-CS (57) was used to compute the European-based polygenic risk score and, separately, the East-Asian-based polygenic risk score for SCZ. Following the recommendation from the program, only SNPs from the HapMap3 reference data were used for PRS computation. Since our sample size was small, the polygenic parameter *phi* was preset to 0.01, i.e., assuming a highly polygenic genetic architecture for SCZ. For the remaining parameters of PRC-CS, the default values were used. The estimated effect sizes from PRS-CS were used to compute PRS for our sample using the *–score* function in PLINK.

### 2.5 Statistical analysis

The relationships of demographic and lifestyle variables with SCZ were assessed individually using *t*-tests (numeric variables: age, BMI, and years of education) or Fisher’s exact test for independence (sex and smoking). BMI was assessed both for the whole sample and in three subgroups: less than 18.49 kg/m^2^; greater than or equal to 18.5 but less than 25.49 kg/m^2^; and greater than or equal to 25.5 but less than or equal to 28 kg/m^2^.

The associations of PRS and α-diversity with SCZ were tested via logistic regression models. The SCZ diagnostic status of subjects was set as the binary dependent variable. PRS was set as the predictor, along with age, sex, years of education, whether the subject had ever smoked, and PC1-10 as covariates. For the association between α-diversity and SCZ, the covariates PC1-10 were replaced by BMI. To estimate the joint effects of PRS and α-diversity indices, both α-diversity indices and PRS were entered into the model, and both PC1-10 and BMI were included as covariates, along with age, sex, years of education, and whether the subject had ever smoked, following the recommendation given by Keller (58). In addition, a term for the interaction between PRS and α-diversity indices was included to investigate whether α-diversity moderates the effects of PRS on SCZ or whether the effects of α-diversity depend on PRS values. Correction for multiple tests was applied using the false discovery rate (59), and corrected *p*-values <0.05 were considered statistically significant. The variance at the observed scale (Nagelkerke-R2) explained by PRS was estimated by first estimating Nagelkerke-R2 explained by the full model and the reduced model, i.e., excluding the focal variable. Next, the difference between the two estimates was used to determine the variance explained by the focal variable. This procedure was performed separately for PRS-EAS and PRS-EUR and for each of the five α-diversity indices.

Linear regression models were used to evaluate the effect of α-diversity on the symptom severity of SCZ patients, as measured by PANSS subscale scores: positive (PANSS-P), negative (PANSS-N), general psychopathology (PANSS-G), and total (PANSS-T). These subscale scores were first scaled to achieve normal distributions and were then used as the dependent variables in the models. Each subscale was analyzed separately. Sex, BMI, years of education, smoking habits, and age were included as covariates in each model. Correction for multiple tests was applied using the false discovery rate (59), and corrected *p*-values <0.05 were considered statistically significant.

To assess the contributions of phylum- and genus-level microbial abundance to α-diversity, linear regression models were used. Here, the transformed diversity indices, except for the Shannon index, were used as dependent variables. The relative abundances of each taxon, separately, were used as predictors, along with the covariates of sex, BMI, years of education, smoking habits, and age. Correction for multiple tests was applied using the false discovery rate (59), and corrected *p*-values <0.05 were considered statistically significant.

As a coarse screen analysis, logistic regressions were used to identify taxa associated with SCZ risk. The SCZ diagnosis status was used as the dependent variable, and the relative abundance was used as a predictor, along with sex, BMI, education years, smoking habits, and age as the model covariates. Correction for multiple tests was applied using the false discovery rate (59), and corrected *p*-values <0.05 were considered statistically significant. Taxa that were associated with SCZ were evaluated for interaction effects with PRS by adding a PRS-by–taxon interaction term to these models. In these models, the covariates were additionally expanded to include the top 10 PCs. Correction for multiple tests was applied using the false discovery rate (59), and corrected *p*-values <0.05 were considered statistically significant.

The PRS-related analyses were performed separately for PRS constructed using GWAS based on East Asian and European samples. Correction for multiple tests was also performed separately for these two sets of analysis.

## 3 Results

### 3.1 Clinical characteristics and data quality

A total of 159 first-episode, drug-naïve SCZ patients and 86 demographically matched HCs were included in the analyses. SCZ patients and HCs did not differ on age, sex, or BMI. For BMI, there was also no effect in each subgroup. Patients had significantly fewer years of education than HCs (*p* = 1.34×10^−4^, Table 1). The rarefaction plot (Supplementary Figure S1) showed that our preprocessing procedure for the microbiome data was reliable. As shown in Supplementary Figures S2, S3, that the transformed α-diversity indices became less skewed and would not be problematic in our statistical analysis.

**Table 1:** Demographic and clinical characteristics of participants.

	SCZ	HCs	Statistics	
			beta	*p*
Sample size (*n*)	159	86	–	–
Sex (M/F)	78/81	35/51	−0.23	0.42
Age (years, mean ± SD)	22.45 ± 7.60	22.93 ± 2.26	−0.009	0.68
PANSS-T	83.49 ± 12.81			
PANSS-P	20.51 ± 4.11			
PANSS-N	21.39 ± 5.69			
PANSS-G	41.58 ± 7.48			
BMI (kg/m^2^, mean ± sd)	21.34 ± 3.80	21.45 ± 2.78	−0.026	0.49
BMI < 18.49	17.39 ± 0.94	17.51 ± 0.61	0.45	0.66
18.5 ≤ BMI < 25.49	21.0 ± 1.74	21.22 ± 1.78	0.81	0.42
25.5 ≤ BMI ≤ 28	26.94 ± 0.63	26.66 ± 0.93	−0.68	0.51
Education (years, mean ± SD)	11.15 ± 2.86	12.36 ± 1.33	−0.223	1.34×10^−4^
Smoking (Yes/No)	6/151	3/83	−0.249	0.75

SCZ, schizophrenia; HCs, healthy controls; BMI, body mass index; PANSS-T/P/N/G, total, positive, negative, and general psychopathology PANSS score; M, male; F, female.

### 3.2 Polygenic risk scores and SCZ

**Figure 1:**
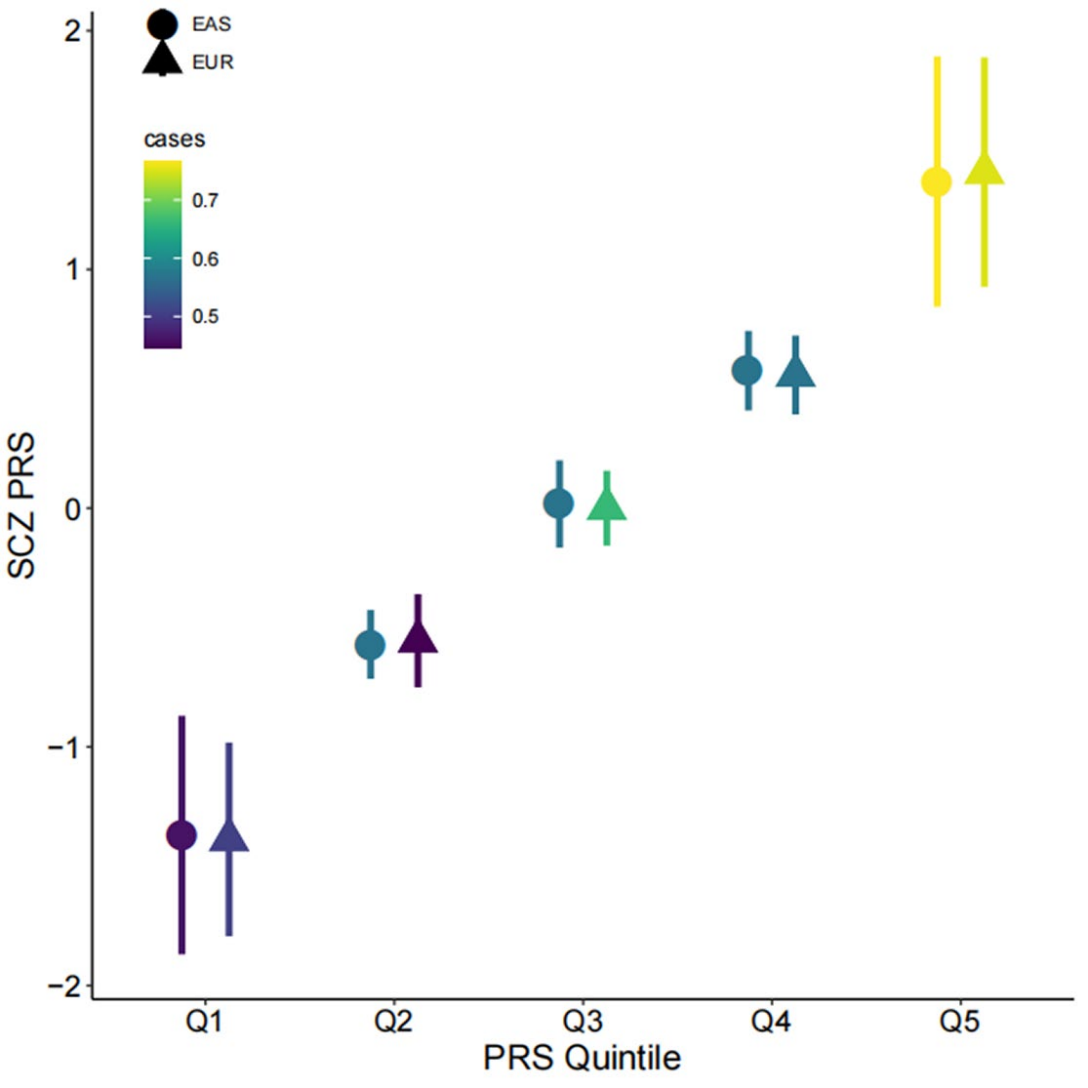
Associations of polygenic risk score with schizophrenia. Participants were divided into quintiles by polygenic risk score (PRS; x-axis, Q1–Q5) for schizophrenia (SCZ) (y-axis). The mean and one standard deviation within each quintile are shown. Colors indicate the proportion of cases in each quintile; the shapes of the symbols indicate whether polygenic scores were computed based on East Asian (EAS) or Caucasian (EUR) GWAS. PRS is shown on a standard deviation scale.

We confirmed significant associations between PRS and the diagnosis in first-episode, drug-naïve patients, with the East-Asian sample-based PRS (PRS-EAS) showing a stronger association (OR = 2.08, 95% CI = 1.51–2.92, *p* = 1.22×10^−5^) than the European sample-based PRS (PRS-EUR) (OR = 1.73, 95% CI = 1.30–2.35, *p* = 2.77×10^−4^). On the observed scale, PRS-EAS and PRS-EUR explained 8.9 and 5.8% of phenotypic variance, respectively. Stratifying participants into quintiles by PRS showed that the proportion of patients increased monotonically from Q1 to Q5 (Figure 1). In the highest stratum (Q5), 82.5 and 78.9% of participants were patients based on PRS-EAS and PRS-EUR, respectively. The corresponding numbers for the lowest stratum (Q1) were 51.7 and 56.9%, respectively. These results suggest that SCZ may have both shared and unique genetic architecture in East Asian and Caucasian populations confirming that matching the ancestry of GWAS samples used to generate a PRS with that of target prediction samples could improve risk prediction.

### 3.3 α-diversity and SCZ

**Figure 2:**
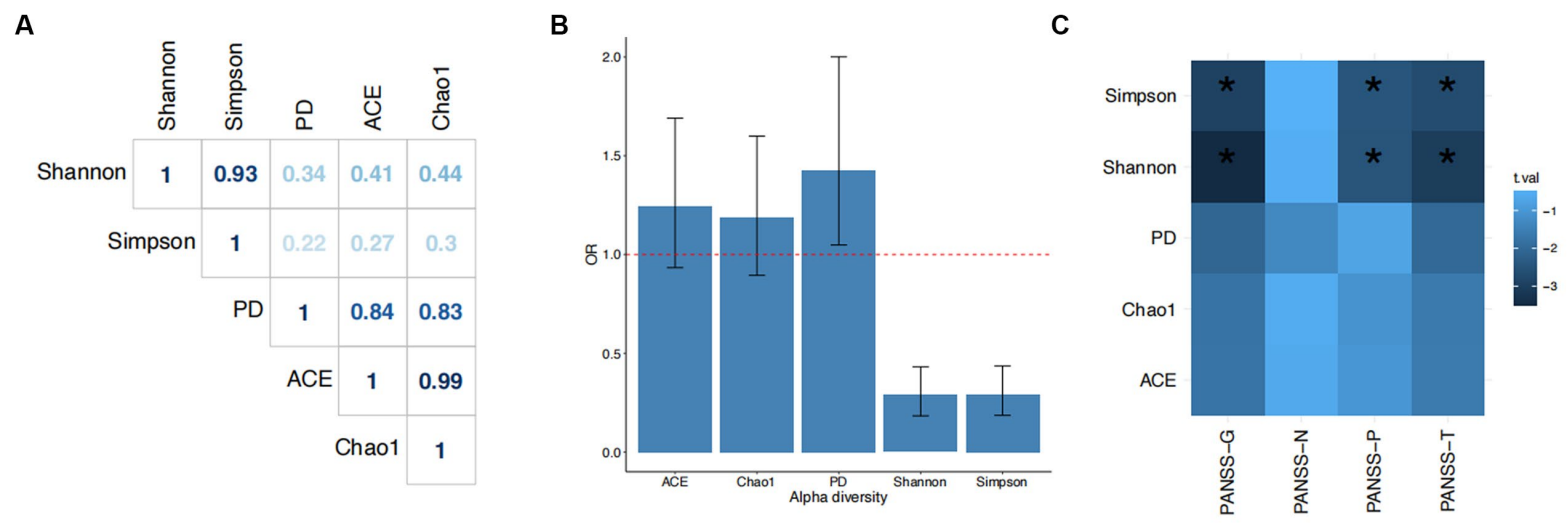
Associations of α-diversity with schizophrenia. **(A)** Pearson correlation coefficients among the five α-diversity indices. **(B)** Odds ratios (ORs) and 95% confidence intervals for the associations of each index with schizophrenia. The dashed line indicates OR = 1. **(C)** Associations between alpha diversity index and PANSS components. Chao1, ACE (Abundance-based Coverage Estimator), PD (Faith’s phylogenetic diversity metric), Shannon, and Simpson are the five alpha diversity indices; PANSS-T, total PANSS score; PANSS-P, PANSS-positive score; PANSS-N, PANSS-negative score; PANSS-G, PANSS-general psychopathology score.

We discovered distinct patterns of association between the five α-diversity indices and SCZ (Figure 2). While all pairwise correlations among the five indices were significant (*p* < 0.05), these indices belong to three groups (Figure 2A). The ACE and Chao1 indices, both of which are richness-based measures, were almost identical (Pearson r = 0.99) but were less correlated to the Shannon and Simpson indices, both of which are evenness-based measures. The latter two showed a significant and strong correlation (Pearson r = 0.93). The phylogenic diversity-based index, PD, sits in between the two groups. The differences between these three groups of indices were also reflected in their associations with the risk of SCZ. An increased score on the Shannon and Simpson indices showed a negative association with SCZ (Shannon: OR = 0.29, 95% CI = 0.18–0.43, *p* = 1.15×10^−8^; Simpson: OR = 0.29, 95% CI = 0.19–0.44, *p* = 1.25×10^−8^). The proportion of phenotypic variance explained by both the Shannon and the Simpson indices was 33%. The PD index showed a weaker association with SCZ (OR = 1.43, 95% CI = 1.05–2.00, *p* = 0.03, Nagelkerke-R2 = 19%). Neither the ACE nor the Chao1 index was associated with the risk of SCZ (Figure 2B).

Moreover, α-diversity was also associated with symptom severity. Both the Shannon and Simpson indices were inversely associated with total, positive, and general psychopathology PANSS scores (*p* < 0.05; PANSS-T: Shannon, beta = −0.31, se = 0.1; Simpson, beta = −0.33, se = 0.09; PANSS-P: Shannon, beta = −0.25, se = 0.1; Simpson, beta = −0.28, se = 0.11; PANSS-G: Shannon, beta = −0.35, se = 0.1; Simpson, beta = −0.33, se = 0.11). Higher diversity as measured by these two indices was associated with lighter symptoms on these three subscales. There were also suggestive associations between PD and PANSS-G (beta = −0.29, se = 0.14) and PANSS-T (beta = −0.28, se = 0.13), but neither of these survived correction for multiple tests. There were no associations between α-diversity and PANSS-negative symptoms.

### 3.4 Interaction between α-diversity and polygenic risk score

To understand whether gut microbial diversity contributes additional information to PRS in predicting SCZ, we entered both diversity index and PRS as predictors in multiple logistic regression models (Materials and Methods section). In general, we observed slightly changed effects for both PRS and the Shannon index compared with the effects estimated via the univariate logistic models. However, all significant predictors from the corresponding univariate models were still significant in the multiple regression models. For example, in the model with the Shannon index and PRS-EAS, the effect of the Shannon index changed from on OR of 0.29 to 0.26, and that of PRS-EAS changed from an OR of 2.08 to 2.27. In the model including both PRS-EAS and the PD index, the effect of PRS-EAS was slightly reduced (OR = 1.97), and the weaker effect of the PD index was reduced to null (*p* = 0.14). In addition, there were no qualitative changes to the effects of PRS-EAS or the Chao1 index in these models compared to those described above. Replacing PRS-EAS with PRS-EUR produced the same pattern. Therefore, the Shannon and Simpson diversity indices may contribute additional information beyond PRS in explaining the risk of SCZ.

**Figure 3:**
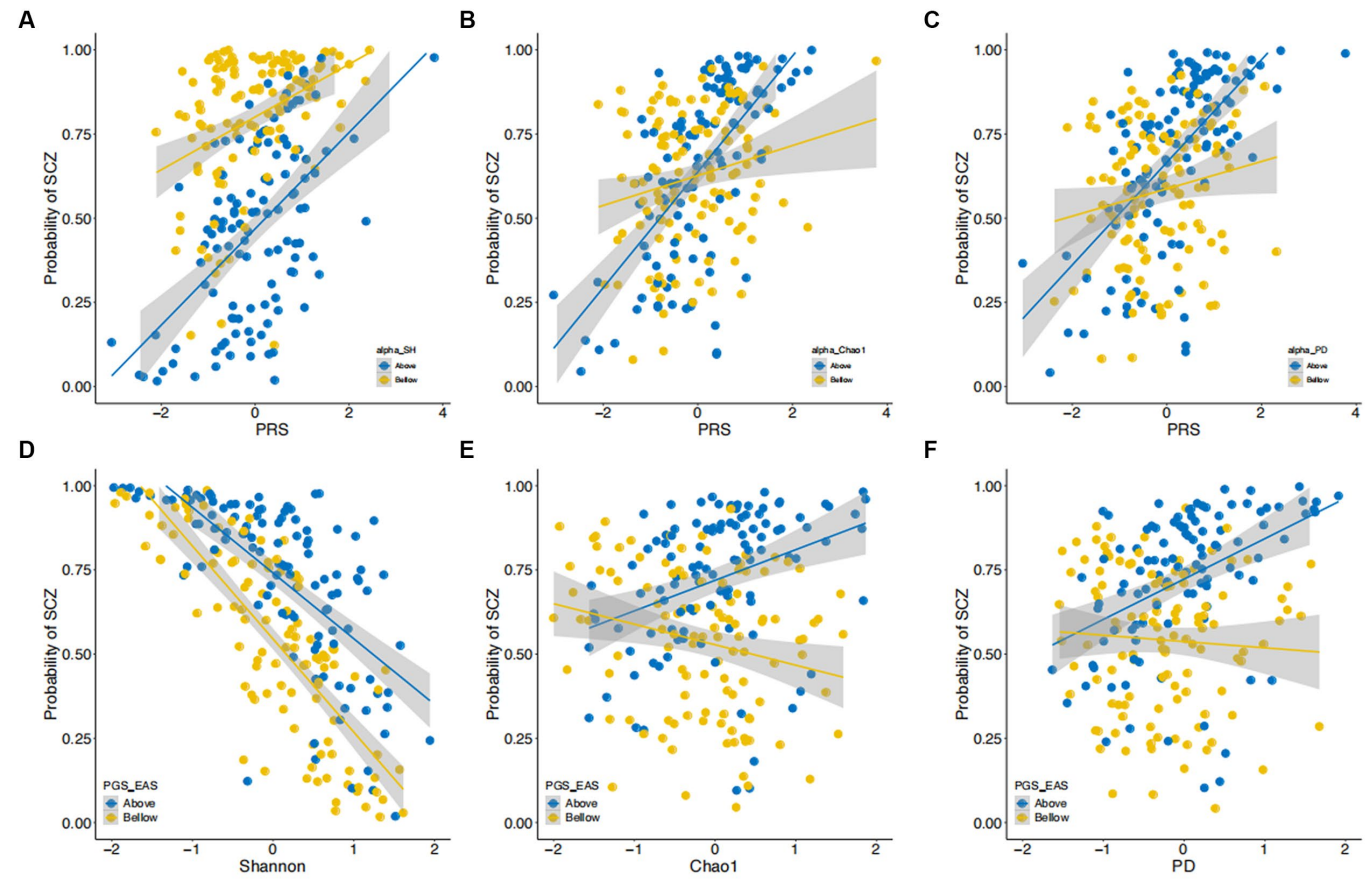
Interactions between α-diversity and polygenic risk scores in their effect on risk of SCZ. **(A–C)** The estimated probability of developing SCZ (y-axis) is plotted against polygenic risk scores (PRS) derived from East Asian GWAS. Data are stratified into two strata (i.e., above and below the median) by Shannon index **(A)**, Chao1 index **(B)**, and PD index **(C)**. **(D–F)** The estimated probability of developing SCZ (y-axis) is plotted against scores on the Shannon **(D)**, Chao1 **(E)**, and PD **(F)** indices. Data are stratified into two strata (i.e., above and below the median) by polygenic risk score (PRS). Both diversity indices and PRS are shown on a standard deviation scale.

Next, we examined whether the effects of α-diversity indices on SCZ were dependent on the genetic risk of participants as measured by PRS. We added terms representing the interactions between α-diversity indices and PRS to the above-described multiple logistic regression models. As illustrated in Figure 3, we found significant interactions between PRS-EAS and the Shannon, Chao1, and PD indices (Shannon, *p* = 0.05; Chao1, *p* = 4.43×10^−3^; and PD, *p* = 6.31×10^−3^). These interaction terms explained 1.05, 3.15, and 2.91% of the phenotypic variance, respectively. There was no interaction effect detected between PRS-EAS and the Simpson index (*p* = 0.11). Interestingly, no significant interaction effects between PRS-EUR and the five α-diversity indices were found.

To illustrate the nature of these significant interaction terms, we stratified our samples by the median values of PRS and, separately, of the α-diversity indices (Figure 3 and Supplementary Figures S4–S7). The effects of PRS-EAS on SCZ risk were larger for participants having higher α-diversity (Figures 3A–C), as measured by the Shannon, Chao1, and PD indices. In other words, when participants were stratified by PRS-EAS, we observed that increasing the α-diversity index measure reduced SCZ risk for participants with lower PRS-EAS. For those with higher PRS-EAS, increasing α-diversity according to the Chao1 and PD indices also increased the SCZ risk (Figures 3D–F). Similar results were also obtained for the Simpson index (Supplementary Figures S4, S5). The ACE index showed similar results to the Chao1 index (Supplementary Figures S6, S7).

To test whether the interaction effect we observed could be due to correlations between PRS-EAS and α-diversity (i.e., the possibility that higher diversity may correlate with lower PRS-EAS), we performed a linear regression analysis of each α-diversity index against PRS-EAS based on HC samples (*N* = 86). In these models, age, sex, BMI, whether the participant had ever smoked, years of education, and PC1-10 were included as covariates. None of the five diversity indices were correlated with PRS-EAS in our sample (Supplementary Table S1). Thus, the effects of PRS-EAS on SCZ seem to interact with gut microbial α-diversity.

### 3.5 Interactions between taxa of the gut microbiota and polygenic score

**Figure 4:**
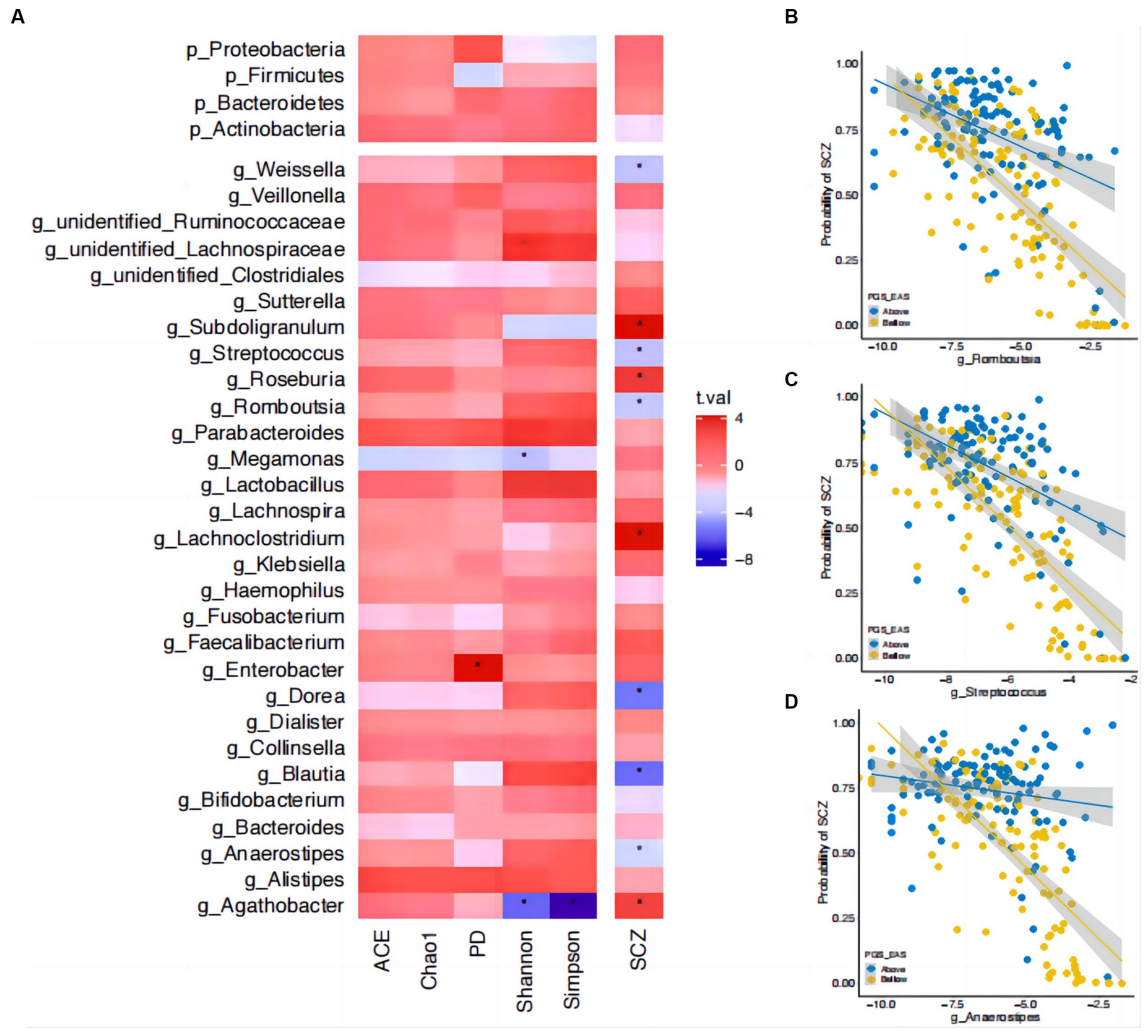
Interactions between microbial abundance and polygenic risk score in their effect on risk of SCZ. **(A)** The associations of gut microbial abundance and alpha diversity index with schizophrenia (SCZ). Asterisks indicate significant associations (corrected *p* < 0.05). The *t*-statistic is indicated by bar color. **(B)** The interactions between polygenic risk score (PRS) and the genus *Romboutsia* in their effect on SCZ risk. **(C)** The interactions between PRS and the genus *Streptococcus* in their effect on SCZ risk. **(D)** The interactions between PRS and the genus *Anaerostipes* in their effect on SCZ risk. The estimated probability of developing SCZ (y-axis) is plotted against the relative abundance of the corresponding genus, with participants stratified into two strata (i.e., above and below the median) on PRS. Microbial relative abundance is shown on a log scale for readability.

To gain a deeper understanding of the observed interaction effects, we tested whether there were interactions between taxon abundance and PRS. Before performing these tests, we first assessed which taxa contributed to α-diversity and SCZ risk (Materials and Methods section). There were four genera contributing to the Shannon index (three genera: *unidentified_Lachnospiraceae*, beta = 0.20, se = 0.05, *p* = 4.43×10^−2^; *Megamonas*, beta = −0.21, se = 0.05, *p* = 1.52×10^−2^; *Agathobacter*, beta = −0.3, se = 0.05, *p* = 1.71×10^−6^), Simpson index (one genus: *Agathobacter*, beta = −0.34, se = 0.04, *p* = 3.51×10^−13^), and PD (one genus: *Enterobacter*, beta = 0.26, se = 0.06, *p* = 6.35×10^−3^) (Figure 4A). No specific taxa contributed to the ACE or Chao1 indices. There were 10 taxa associated with SCZ risk, among which six showed a protective effect, i.e., higher abundance was associated with a lower risk, and four showed a negative effect (Figure 4A and Supplementary Table S2). While there was no interaction effect between the genus *Agathobacter* (which was associated with both α-diversity and SCZ) and PRS, three genera showed significant interactions with PRS in their effect on SCZ risk (Figures 4C,D): *Romboutsia* (interaction beta = 0.84, se = 0.3, *p* = 0.03), *Streptococcus* (interaction beta = 0.89, se = 0.34, *p* = 0.03), and *Anaerostipes* (interaction beta = 1.37, se = 0.51, *p* = 0.03). For these three genera, increased abundance was associated with greater protective effects against SCZ in individuals with a lower-than-median PRS than those having above-median PRS (Figures 4C,D). We obtained qualitatively the same interaction effects between these three genera and PRS-EUR (Supplementary Table S3).

## 4 Discussion

Gut commensal microbes can contribute to the host physiology by producing beneficial metabolites, such as short fatty acids, neurotransmitters, and proinflammatory factors (60). Thus, species-rich gut ecosystems are more robust to insults originating from surrounding environments or from the host genome (61). Here, we show that the strengths of such protective effects depend on the host’s genetic risk for a disease, e.g., SCZ. For genetically predisposed individuals (with a high PRS), increasing the α-diversity of the gut microbiota (as measured, e.g., by the Shannon index) provided less protection than for those with low PRS. The pattern of interactions also varied with the way in which α-diversity was quantified. The species richness-dominant measures (Chao1, ACE, and PD) (32, 39) showed effects in opposite directions for genetically high- and low-risk individuals. This may indicate that rare bacteria—which dominate in the Chao1, ACE, and PD indices, but not in the Shannon and Simpson indices—in the guts of such individuals not achieved commensal status with the host. At the genus level, we found interaction effects between PRS and the relative abundance of *Romboutia*, *Streptococcus,* and *Anaerostipes*. Importantly, these genus-level interaction effects were also observed when the European sample-based GWAS was used to construct PRS (Supplementary Table S3), further supporting these microbiota interaction effects. Nevertheless, these novel findings call for replication in independent large-scale studies.

Our results confirmed a polygenic architecture for first-episode, drug-naïve SCZ patients. Most previous studies have tested the predictive value of PRS in chronic SCZ patients (6, 8), where disease duration, use of medications, and other environmental factors may reduce predictive performance. Despite the small sample size, our data showed that PRS have a significant discriminatory power in these drug-naïve patients. Our estimates for the proportion of phenotypic variance explained by PRS are within the range reported by previous studies with chronic patients (6, 8). PRS for our sample, built on both ancestrally matched East Asian GWAS and European GWAS results, were significantly associated with SCZ. In line with a previous report (62), ancestrally matched PRS showed stronger predictive performance for our sample than unmatched PRS. The hypothesis that differences in allele frequencies between the two populations may contribute to this cross-ancestral difference in predictions (8) is also supported by our data (Supplementary Figure S8).

The large effects of PRS on SCZ in our sample allowed us to study the interactions of this factor with gut microbiota features. Our findings may serve as a novel explanation for the conflict results on the relationship between α-diversity and SCZ. While α-diversity as measured by the Chao1 and Shannon indices has been shown to be lower in SCZ patients than in HCs in several studies (24, 27, 30, 63, 64), others have reported null findings (65–68). Moreover, one study has reported observing a higher Shannon index in patients compared to healthy controls (69). In the present study, our findings suggested that the Shannon and Simpson indices were lower in SCZ patients, but we did not find significant associations for the other richness-dominant indices. We further showed that scores on the Simpson and Shannon indices were also associated with disease severity as measured by PANSS scores. It is well known that medical treatments and disease duration have a major impact on the composition of the gut microbiota. Not all previous studies have been performed with drug-naïve patients (24, 63). Our observed associations are in line with a recent review article, in that α-diversity has been found to be lower compared to healthy controls in drug-naïve patients but not in chronic patients (38). In addition, our results are based on a larger sample than previous microbiome studies, thus higher statistical power was expected. The interaction between PRS and α-diversity in terms of their effects on SCZ showed that the effects of scores on the Chao1, Shannon, and PD indices may depend on the individual’s genetic risk score for SCZ. Therefore, it is conceivable that previous studies may have included patients with low genetic risk but high environmental contributions. One possible explanation for the interaction effects may be that high genetic risk for SCZ may alter the gut ecosystem in such a way that fewer microbes are able to colonize the gut. For individuals with low genetic risk of SCZ, the beneficial effect of increased scores on the Chao1, ACE, and PD indices also showed a smaller protective effect than increased scores on the Shannon and Simpson indices. Thus, future studies with larger samples and controlling for the genetic composition of the host (such as via PRS) may resolve these complex relationships.

We attempted to further explain the interaction between PRS and diversity by studying the PRS–genus interactions. The results are intriguing. While we expected to observe some interaction effects for the genus that contributed to both α-diversity and SCZ, i.e., *Agathobacter*, this was not the case. A relatively high abundance of *Agathobacter* was associated with a positive effect on SCZ, but this genus was negatively correlated with scores on the Shannon and Simpson indices, and there were no interaction effects. We then screened for interactions between genera that were associated with SCZ in our sample and PRS. Among the three identified interacting genera, *Streptococcus*, which is involved in the metabolism of lactic acid, glutamate, and GABA, has been reported to be associated with SCZ in previous studies (64, 65), but the directions of effects observed were opposing. The other genus, *Anaerostipes*, has been reported to have a lower abundance in SCZ (66), which is congruent with our findings here. The last genus, *Romboutsia*, was very recently characterized in 2014 (70) and thus has not been reported to be associated with any psychiatric conditions. However, a recent large population study has suggested that *Romboutsia* may have a health-promoting effect via production of short-chained fatty acids (71). Of note, these interactions with PRS built on East Asian GWAS were also statistically significant for the PRS built on GWAS from a European sample, further supporting these interactions between genetics and the microbiota in schizophrenia.

The present study has both strengths and weaknesses. We removed the effects of antipsychotic treatments and disease duration on gut microbiota composition by focusing on first-episode, drug-naïve SCZ patients. Due to the challenge of recruiting drug-naïve patients, our sample size is small compared to that of a typical genetic study. Thus, we expect that future larger-scale studies will replicate and extend our findings here. A major limitation is that we did not collect data on lifestyle and other environmental variables, such as prenatal life factors, childhood adverse effects, diet, physical activity, and migrations, all of which have been shown to be potential confounders in associations between the microbiota and schizophrenia (33). This is a common problem in most previous studies (33, 72). We additionally applied a genetics-based correction method (73) by rerunning our analysis with the top 10 PCs included as additional covariates. The results were qualitatively unchanged compared to our main analysis. Moreover, we only analyzed data from gut commensal bacteria and the effects of aggregated DNA variations. Future work should investigate the effects of other commensal microorganisms (e.g., fungi) (74) and epigenetic variations (75, 76). Nevertheless, these limitations must be taken into account in the interpretation of our findings here. We expect that future studies with well-controlled confounders will confirm our results.

Here, as a proof of principle, we show that integrating PRS and gut microbiome research can provide novel insights into the complex interplay of these factors in SCZ and may form the foundation for the development of probiotic treatments for SCZ (77–79). Our findings also indicate that stratifying samples on the basis of gut microbial composition may improve prediction of the risk of SCZ and provide new insights into the “missing heritability” (80) problem in SCZ. In conclusion, our findings show that both gut microbial markers and host genetics interactively contribute to the risk of SCZ.

## Data availability statement

SCZ GWAS summary statistics for both European and East Asian populations were downloaded from https://www.med.unc.edu/pgc/download-results/scz/. The raw genotype datasets presented in this article are not readily available because of local regulations. The raw data of microbiome that supporting the conclusions of this article will be made available by the authors, without undue reservation.

## Ethics statement

The studies involving humans were approved by the Human Ethics Committee of The First Affiliated Hospital of Zhengzhou University (no. 2016-LW-17). The studies were conducted in accordance with the local legislation and institutional requirements. Written informed consent for participation in this study was provided by the participants’ legal guardians/next of kin.

## Author contributions

LZ: Data curation, Formal analysis, Resources, Visualization, Writing – original draft, Writing – review & editing. XY: Data curation, Resources, Writing – review & editing. XL: Resources, Writing – review & editing. XZ: Data curation, Resources, Writing – review & editing. YM: Formal analysis, Methodology, Writing – review & editing. SH: Supervision, Writing – review & editing. OA: Funding acquisition, Supervision, Writing – review & editing. YW: Conceptualization, Funding acquisition, Supervision, Visualization, Writing – original draft, Writing – review & editing. XS: Conceptualization, Funding acquisition, Project administration, Resources, Supervision, Writing – review & editing.
