# Supplementary material for: Gut microbial diversity moderates polygenic risk of schizophrenia
Source: Front Psychiatry. 2024 Feb 1;15:1275719. doi: 10.3389/fpsyt.2024.1275719 (PMC10868137; doi:10.3389/fpsyt.2024.1275719)
Supplement: Supplementary file 1 [file Data_Sheet_1.docx]

**Supplementary Figures**

**Figure S1. Rarefaction curves.**

**a**


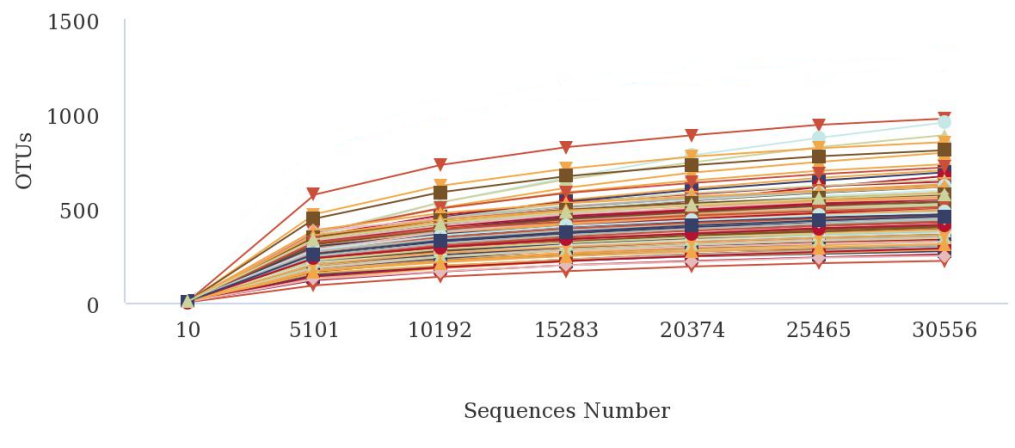


**b**


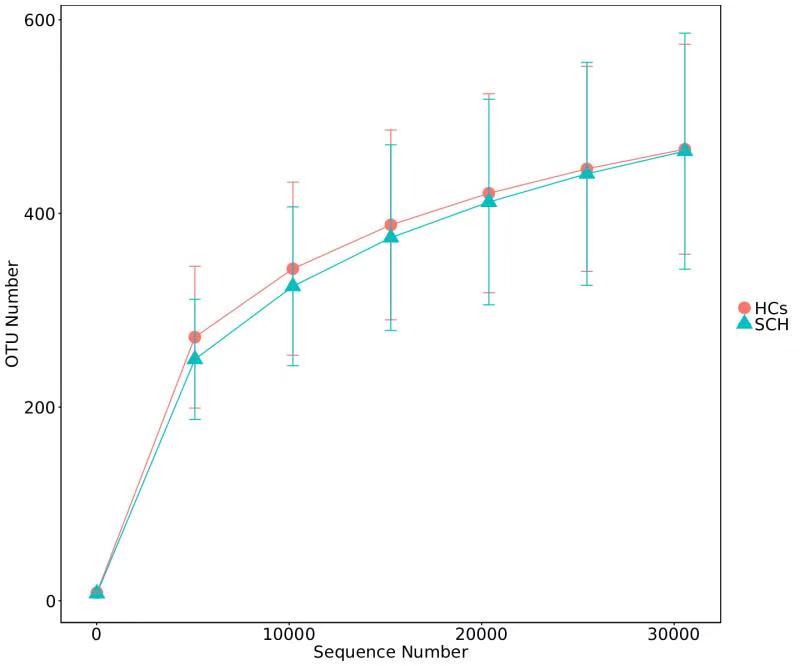


The majority of rarefaction curves tended to approach the saturation plateau, suggesting the sequencing depth of the gut microbiome was sufficient.

**Figure S2. Distributions for alpha diversity before transformation.**

**Figure S3. Distributions for alpha diversity after transformation.**

ACE index was first log-transformed and then scale to have mean 0 and standard deviation 1; Chao1 index was first log-transformed and then scale to have mean 0 and standard deviation 1; PD index was first log-transformed and then scale to have mean 0 and standard deviation 1; Shannon index was scale to have mean 0 and standard deviation 1; Simpson index was logit transformed.

**Figure S4. Association of polygenic risk scores with SCZ stratified by the Simpson index.**

The estimated probabilities of developing SCZ (y axis) were plotted against polygenic risk scores (PRS) derived from East Asian GWAS. Data were stratified by Simpson index into two strata, i.e., above and below the median.

**Figure S5. Association of Simpson index with SCZ stratified by polygenic risk scores.**

The estimated probabilities of developing SCZ (y axis) were plotted against Simpson index. Data were stratified by polygenic risk scores (PRS) derived from East Asian GWAS into two strata, i.e., above and below the median. The Simpson diversity index was shown on standard deviation scale.

**Figure S6. Association of polygenic risk scores with SCZ stratified by ACE index.**

The estimated probabilities of developing SCZ (y axis) were plotted against polygenic risk scores (PRS) derived from East Asian GWAS. Data were stratified by ACE index into two strata, i.e., above and below the median.

**Figure S7. Association of ACE index with SCZ stratified by polygenic risk scores.**

The estimated probabilities of developing SCZ (y axis) were plotted against ACE index. Data were stratified by polygenic risk scores (PRS) derived from East Asian GWAS into two strata, i.e., above and below the median. The ACE diversity index was shown on standard deviation scale.

**Figure S8. Correlations of SNP minor allele frequencies between our sample and EUR sample.**


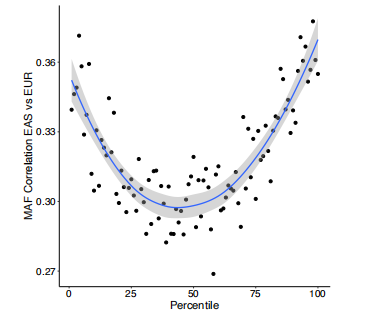


European sample based GWAS Posterior effect sizes estimated by PRS-CS1 were divided into 100 percentiles (x axis). The mean correlation in each percentile between the minor allele frequencies in the 1000 Genomes2 EUR sample and in our Chinese sample were plotted, and confidence band were estimated by the *loess* function from R.

# Ge T, Chen C-Y, Ni Y, Feng Y-CA, Smoller JW. Polygenic prediction via Bayesian regression and continuous shrinkage priors. *Nature Communications* 2019; **10**(1)**:** 1776.

1. Auton A, Brooks LD, Durbin RM, Garrison EP, Kang HM, Korbel JO *et al.* A global reference for human genetic variation. *Nature* 2015; **526**(7571)**:** 68-74.

**Figure S9. Posterior effect sizes estimated by PRS-CS.**

European sample based GWAS Posterior effect sizes estimated by PRS-CS^1^ were divided into 100 percentiles (x axis). This figure shows that near the 50^th^ percentile, the effect sizes are close to null.

1. Ge T, Chen C-Y, Ni Y, Feng Y-CA, Smoller JW. Polygenic prediction via Bayesian regression and continuous shrinkage priors. *Nature Communications* 2019; **10**(1)**:** 1776.

Table S1. PRS-EAS vs alpha diversity correlations in healthy control group.

|  | Beta | p |
| --- | --- | --- |
| Shannon | -0.18 | 0.158 |
| Simpson | -0.19 | 0.136 |
| Chao1 | -0.20 | 0.053 |
| ACE | -0.17 | 0.098 |
| PD | -0.11 | 0.318 |

Each of the five indices was regressed on PRS-EAS, including age, gender, BMI, smoking and PC-10 as covariates. Both the index and PRS-EAS were standardized to have mean 0 and standard deviation 1. The coefficients of PRS-EAS (beta) and p values (p) were shown in the table.

Table S2. Taxa associations with SCZ risk.

| Taxa | Beta | Se | zval | pval | FDR |
| --- | --- | --- | --- | --- | --- |
| g_Bacteroides | -0.155957362051905 | 0.134435009173454 | -1.16009485186022 | 0.246010189977482 | 0.386587441393185 |
| g_Megamonas | 0.128887493014448 | 0.153306123356426 | 0.840719797700405 | 0.40050492687922 | 0.495940432773269 |
| g_Agathobacter | 0.548737514533507 | 0.185051094917088 | 2.96532973652152 | 0.00302358661203194 | 0.00997783581970541 |
| g_Subdoligranulum | 1.32256133564272 | 0.31486868323226 | 4.2003584544073 | 2.66492730209156e-05 | 0.000279588025364551 |
| g_Faecalibacterium | 0.313202364606594 | 0.148997744278205 | 2.10206111591723 | 0.0355479224957572 | 0.0977567868633322 |
| g_unidentified_Clostridiales | -0.0316961813404968 | 0.130687705816034 | -0.242533765074388 | 0.808366596937306 | 0.808366596937306 |
| g_Alistipes | -0.146282682048635 | 0.152230468687654 | -0.960929065710077 | 0.336587835203724 | 0.482930372248821 |
| g_Blautia | -1.51803846621394 | 0.265056253484222 | -5.72723128112991 | 1.02082954883081e-08 | 3.36873751114169e-07 |
| g_Roseburia | 0.729405307991098 | 0.225582824730372 | 3.23342572229478 | 0.00122315138732785 | 0.00504549947272736 |
| g_unidentified_Ruminococcaceae | -0.193059832932234 | 0.135196325299597 | -1.42799615673289 | 0.153292964733975 | 0.281037102012288 |
| g_Bifidobacterium | -0.35280022349844 | 0.161609005143243 | -2.18304804974038 | 0.0290322717068279 | 0.0870968151204837 |
| g_Dialister | -0.0505009305277082 | 0.136943503085041 | -0.36877200736093 | 0.712297671250432 | 0.770843814136365 |
| g_Lachnospira | 0.286430899064601 | 0.216643846660617 | 1.32212801554114 | 0.186125526225144 | 0.316839253350851 |
| g_unidentified_Lachnospiraceae | -0.238103036254148 | 0.134289111872905 | -1.77306285620159 | 0.0762182919653098 | 0.167680242323682 |
| g_Enterobacter | 1.01578774095791 | 0.686556187488583 | 1.47954058162909 | 0.138995893613784 | 0.269815558191462 |
| g_Romboutsia | -1.00841714704566 | 0.269534298267941 | -3.74133145030472 | 0.000183047921368928 | 0.000862940200739232 |
| g_Lachnoclostridium | 1.45830755978936 | 0.351770627772616 | 4.14562059664633 | 3.38894576199455e-05 | 0.000279588025364551 |
| g_Parabacteroides | -0.10940044100083 | 0.131591902912235 | -0.831361493980325 | 0.405769444996311 | 0.495940432773269 |
| g_Klebsiella | 0.972099388529125 | 0.745123256429726 | 1.30461555204566 | 0.192023789909607 | 0.316839253350851 |
| g_Veillonella | 0.488495612292087 | 0.527492352425842 | 0.926071458753067 | 0.354408845322464 | 0.487312162318388 |
| g_Fusobacterium | -0.0393332279579953 | 0.134980604499704 | -0.291399109551932 | 0.77074609548764 | 0.794831910971628 |
| g_Anaerostipes | -0.763483767401577 | 0.242468286946183 | -3.14879845532556 | 0.00163943220012656 | 0.00601125140046406 |
| g_Lactobacillus | -0.0984306057453771 | 0.132690278148706 | -0.741807215409299 | 0.458204148271431 | 0.540026317605615 |
| g_Streptococcus | -1.29127411313969 | 0.328554841331718 | -3.93016309820858 | 8.48882602291293e-05 | 0.000502573671688842 |
| g_Dorea | -1.70304345569014 | 0.315392865682803 | -5.39975262916353 | 6.67328498022815e-08 | 1.10109202173764e-06 |
| g_Sutterella | 0.711442490911354 | 0.393542196931411 | 1.80779214137321 | 0.0706388573331689 | 0.166505877999612 |
| g_Weissella | -5.05305421689409 | 1.29154292571613 | -3.91241678172818 | 9.13770312161531e-05 | 0.000502573671688842 |
| g_Haemophilus | -0.423371968952271 | 0.260322692340876 | -1.62633524240711 | 0.103878358523751 | 0.214249114455237 |
| g_Collinsella | -0.127375282952983 | 0.143833812575758 | -0.885572597096349 | 0.375847883472269 | 0.495940432773269 |
| p_Firmicutes | 0.0700732722968616 | 0.136468859763286 | 0.513474446979394 | 0.60761948090377 | 0.691429064476704 |
| p_Bacteroidetes | -0.0484478366985705 | 0.137265481478306 | -0.352949890801406 | 0.724126007219009 | 0.770843814136365 |
| p_Proteobacteria | 0.191231300770919 | 0.184752792362283 | 1.0350658213378 | 0.300638172998025 | 0.450957259497037 |
| p_Actinobacteria | -0.310705996426875 | 0.151645024858255 | -2.0489033301112 | 0.0404715686522138 | 0.10273552042485 |

Table S3 Interaction effect of taxon and EUR_PRS.

| Taxa | Beta | Se | tval | pval | FDR |
| --- | --- | --- | --- | --- | --- |
| g_Bacteroides | 0.0766748051708944 | 0.0706133836079487 | 1.08583955694007 | 0.27874922237008 | 0.629911886287059 |
| g_Megamonas | 0.0787895228890814 | 0.0710802716534742 | 1.10845838171794 | 0.2688853057782 | 0.629911886287059 |
| g_Agathobacter | -0.0447807421824619 | 0.0721059258156957 | -0.621041081934409 | 0.535221392344087 | 0.933302235315662 |
| g_Subdoligranulum | 0.0413924933633978 | 0.0719450818704176 | 0.575334578643625 | 0.565658395310392 | 0.933302235315662 |
| g_Faecalibacterium | 0.107137950280185 | 0.07052549282546 | 1.51913791719734 | 0.13017673039983 | 0.536979012899298 |
| g_unidentified_Clostridiales | 0.0321448115178637 | 0.072608967976367 | 0.442711312579547 | 0.658413512764355 | 0.933302235315662 |
| g_Alistipes | 0.0218772979022434 | 0.0726533787793807 | 0.301118795433809 | 0.763610919803723 | 0.933302235315662 |
| g_Blautia | -0.194956558097628 | 0.0643022002577305 | -3.03188004945741 | 0.00272470310986416 | 0.0899152026255174 |
| g_Roseburia | -0.0283316053902951 | 0.0701974565213799 | -0.403598745513895 | 0.686903407397904 | 0.933302235315662 |
| g_unidentified_Ruminococcaceae | 0.076642173945213 | 0.0716684050485271 | 1.06939974307114 | 0.286072239597314 | 0.629911886287059 |
| g_Bifidobacterium | -0.0144484841337394 | 0.0730331446720562 | -0.197834616031091 | 0.843358811625194 | 0.959684164952807 |
| g_Dialister | 0.123724554161427 | 0.070813234830105 | 1.74719534361433 | 0.0820116347852838 | 0.49334825977605 |
| g_Lachnospira | -0.140469478932628 | 0.0704255285727044 | -1.99458181968224 | 0.0473341024998594 | 0.49334825977605 |
| g_unidentified_Lachnospiraceae | -0.110473522001769 | 0.0698570425238085 | -1.581422831694 | 0.115231137039896 | 0.536979012899298 |
| g_Enterobacter | 0.0821772160812456 | 0.0730481985717082 | 1.12497252071967 | 0.261837758965105 | 0.629911886287059 |
| g_Romboutsia | -0.0417441632069479 | 0.0687232910244299 | -0.607423809085461 | 0.544201876933296 | 0.933302235315662 |
| g_Lachnoclostridium | 0.0364839574703813 | 0.0712143422710588 | 0.512311934743631 | 0.608951359453871 | 0.933302235315662 |
| g_Parabacteroides | -0.0237559081915589 | 0.0729671693968598 | -0.325569819795987 | 0.74506212104725 | 0.933302235315662 |
| g_Klebsiella | 0.099760278904688 | 0.0713793652304958 | 1.39760669743343 | 0.163651832619527 | 0.560330382984416 |
| g_Veillonella | -0.0285726934677711 | 0.0699075155899274 | -0.408721340283018 | 0.683145388111749 | 0.933302235315662 |
| g_Fusobacterium | -0.0225034311098677 | 0.0723222697841343 | -0.31115493439345 | 0.755980172409192 | 0.933302235315662 |
| g_Anaerostipes | -0.129726932509554 | 0.0720750205776025 | -1.79988755424465 | 0.0732610041413996 | 0.49334825977605 |
| g_Lactobacillus | 0.0756354691544336 | 0.0707640304533487 | 1.0688406054584 | 0.286323584675936 | 0.629911886287059 |
| g_Streptococcus | -0.0966849823848158 | 0.0701931622670413 | -1.37741311635155 | 0.169797085752853 | 0.560330382984416 |
| g_Dorea | 0.00719636990788422 | 0.0729414289128565 | 0.0986595685763403 | 0.921499229309056 | 0.984398934798065 |
| g_Sutterella | 0.147214985459786 | 0.0725177409070617 | 2.03005476478447 | 0.0435657077653672 | 0.49334825977605 |
| g_Weissella | -0.118702851088352 | 0.0696380798593549 | -1.70456812318909 | 0.0896996835956455 | 0.49334825977605 |
| g_Haemophilus | -0.050669218725086 | 0.072467225540059 | -0.699201857770541 | 0.485171220135766 | 0.933302235315662 |
| g_Collinsella | -0.0529001338664739 | 0.0682318954923682 | -0.775299198193767 | 0.439003066083653 | 0.905443823797534 |
| p_Firmicutes | -0.00294951483392224 | 0.0705555818590252 | -0.0418041316676485 | 0.966693103575088 | 0.984398934798065 |
| p_Bacteroidetes | 0.00544572616286241 | 0.0705389945444512 | 0.0772016414187858 | 0.938533944339022 | 0.984398934798065 |
| p_Proteobacteria | 0.00142081856497637 | 0.0725769182595505 | 0.0195767276848987 | 0.984398934798065 | 0.984398934798065 |
| p_Actinobacteria | -0.0168696709521223 | 0.0728069911800739 | -0.23170399818334 | 0.816985271067298 | 0.959684164952807 |
